# Data on water quality index for the groundwater in rural area Neyshabur County, Razavi province, Iran

**DOI:** 10.1016/j.dib.2017.10.052

**Published:** 2017-11-01

**Authors:** Mahmood Yousefi, Hossein Najafi Saleh, Ali Akbar Mohammadi, Amir Hossein Mahvi, Mansour Ghadrpoori, Hamed Suleimani

**Affiliations:** a Students Research Committee, Neyshabur University of Medical Sciences, Neyshabur, Iran; b Torbat Heydarieh University of Medical Sciences, Torbat Heydarieh, Iran; c Department of Environmental Health Engineering, Neyshabur University of Medical Sciences, Neyshabur, Iran; d School of Public Health, Tehran University of Medical Sciences, Tehran, Iran; e Center for Solid Waste Research, Institute for Environmental Research, Tehran University of Medical Sciences, Tehran, Iran; f Department of Environmental Health Engineering, Faculty of Health and Nutrition, Lorestan University of Medical Sciences, Khorramabad, Iran

**Keywords:** Ground water quality index, Rural area, Neyshabur, Iran

## Abstract

Public health is at risk from physical and chemical contaminants in the drinking water which may have immediate health consequences. The data from the current study was evaluated for groundwater quality in the rural villages of Neyshabur County in Iran. For determination of the essential physicochemical parameters, water samples were collected from 30 randomly-selected water wells during 2013 and 2014. The samples were tested in situ to measure physical parameters of pH and electrical conductivity and chemical parameters of total dissolved solids, total hardness and levels of calcium, magnesium, carbonates, bicarbonates, sodium, potassium, chloride and sulfates. The APHA method was applied to determine the physicochemical parameters of the water samples.

**Specifications Table**

**Table:** 

Subject area	Chemistry
More specific subject area	Describe narrower subject area
Type of data	Table and figure
How data was acquired	All experiments were done using titrimetric testing for temporary and permanent hardness, calcium, magnesium and chloride. System testing also included pH (WTW model) and electrical conductivity (ESI model). The analysis of sulfate anions and cations was done by spectrophotometry (DR 5000; Hach) in water. The total hardness and TDS were determined by the EDTA titrimetric method and gravimetry, respectively.
Data format	Raw, analyzed
Experimental factors	All water samples were stored in polyethylene bottles in a dark place at room temperature.
Experimental features	
Data source location	Neyshabour, Razavi Khorasan Province, Iran
Data accessibility	Data are included in this article and supplement file excel

**Value of data**

• Determination of the levels of the physical and chemical parameters of EC, pH, TDS, TH, Ca, Mg, CO3, HCO3, Na, K, Cl and SO4 in groundwater in the rural villages of Neyshabur county in Iran.
• The result of analysis of the data shows that the water in this area is not desirable for drinking.
• The levels of SSP, Na and TH were high during both years, indicating that most of the groundwater locations were not suitable for irrigation purposes.

## 1 Data

**Fig. 1:**
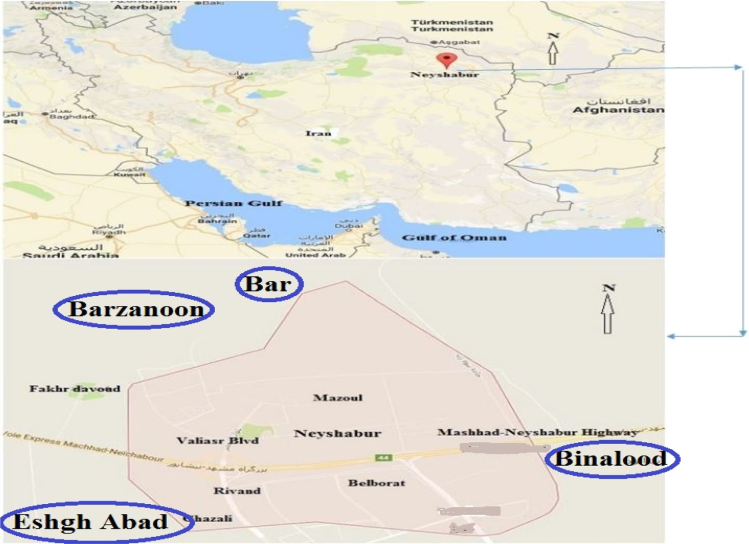
The map and location of sampling villages.

The data was collected for analysis of the physical and chemical parameters of pH, EC, TDS, HCO_3_, CO_3_, SO_4_, Cl, Ca, Mg and Na in the groundwater of Neyshabur county in Iran. Fig. 1 shows the study area and the sampling points. A summary of water quality characteristics is presented in Table 1, Table 2, Table 3. The results for groundwater quality are presented in Table 4. The classification of groundwater samples for use in irrigation based on the results for EC, SAR, RSC, KR, SSP, PI, MH, Na and TH is presented in Table 5.

**Table 1:** Water level and physicochemical analyses of ground water samples of study area collected during 2013.

**Well**	**pH**	**Na**^**+**^	**Mg**^**+2**^	**Ca**^**+2**^	**Cl**^**−**^	**K**^**+**^	**CO3**^**−2**^	**HCO3**^**−**^	**SO4−2**	**TDS**	**EC**	**T.H**
**no**		**(mg/l)**	**(mg/l)**	**(mg/l)**	**(mg/l)**	**(mg/l)**	**(mg/l)**	**(mg/l)**	**(mg/l)**	**(mg/l)**	**(μmhos/cm)**	**(mg/L)**
P1	8	75.9	18.15	20	28.4	0	0	164.7	115.2	346.5	550	125
P2	7.9	124.2	65.34	80	173.95	0	0	237.9	297.6	907.2	1440	470
P3	8.2	266.8	29.04	24	191.7	0	0	244	283.2	945	1500	180
P4	7.8	319.7	36.3	86	436.65	0	0	85.4	355.2	1341.9	2130	365
P5	7.8	319.7	20.57	54	326.6	0	0	79.3	374.4	1152.9	1830	220
P6	7.9	271.4	50.82	106	401.15	0	0	256.2	288	1278.9	2030	475
P7	7.8	195.5	32.67	96	276.9	0	0	207.4	240	974.61	1547	375
P8	7.6	920	116.16	304	1562	85.8	0	262.3	902.4	4151.7	6590	1240
P9	7.7	740.6	188.76	184	1100.5	70.2	0	183	1200	3628.8	5760	1240
P10	7.7	1288	111.32	256	2130	144.3	0	195.2	897.6	5040	8000	1100
P11	7.9	722.2	96.8	140	1153.8	23.4	0	91.5	624	2961	4700	750
P12	8.2	423.2	41.14	72	550.25	0	0	109.8	379.2	1600.2	2540	350
P13	7.8	1127	104.06	292	1952.5	117	0	103.7	897.6	4630.5	7350	1160
P14	7.9	1173	104.06	280	1988	120.9	0	97.6	926.4	4725	7500	1130
P15	8.2	724.5	104.06	88	869.75	23.4	0	231.8	816	2709	4300	650
P16	8.1	347.3	33.88	64	436.65	0	0	170.8	297.6	1304.1	2070	300
P17	8.2	821.1	67.76	74	940.75	0	0	183	748.8	2822.4	4480	465
P18	8	142.6	41.14	58	142	0	0	274.5	201.6	737.1	1170	315
P19	8.2	338.1	48.4	46	525.4	0	0	140.3	187.2	1310.4	2080	315
P20	8.8	379.5	6.05	6	326.6	0	18	183	211.2	1088.6	1728	40
P21	8	1150	113.74	172	1863.8	136.5	0	91.5	849.6	4422.6	7020	900
P22	8.1	724.5	104.06	114	1065	23.4	0	115.9	696	2923.2	4640	715
P23	7.9	1564	113.74	272	2591.5	183.3	0	67.1	1046.4	5922	9400	1150
P24	9	1281.1	44.77	26	1295.8	120.9	60	317.2	974.4	3937.5	6250	250
P25	8.2	676.2	93.17	146	1207	15.6	0	225.7	331.2	2822.4	4480	750
P26	8	20.7	21.78	50	28.4	0	0	170.8	86.4	298.62	474	215
P27	8.4	75.9	14.52	24	42.6	0	24	140.3	76.8	335.16	532	120
P28	8.4	43.7	21.78	36	31.95	0	12	201.3	48	321.3	510	180
P29	8.2	285.2	16.94	52	319.5	0	0	152.5	235.2	1030.1	1635	200
P30	8.1	18.4	19.36	36	24.85	0	0	158.6	48	245.7	390	170
**Min**	7.6	18.4	6.05	6	24.85	0	0	67.1	48	245.7	390	40
**Max**	9	1564	188.76	304	2591.5	183.3	60	317.2	1200	5922	9400	1240
**Ave**	8.07	552	62.68	108.6	799.5	35.49	3.8	171.41	487.84	2197.1	3487.5	530.5
**SD**	0.30	445.21	44.36	89.89	738.5	56.44	12.05	65.84	351.13	1683.1	2671.57	386.23

**Table 2:** Water level and physico-chemical analyses of ground water samples of study area collected during 2014.

**Well**	**pH**	**Na**^**+**^	**Mg**^**+2**^	**Ca**^**+2**^	**Cl**^−^	**K**^**+**^	**CO3**^−**2**^	**HCO3**^−^	**SO4**^−**2**^	**TDS**	**EC**	**T.H**
**no**		**(mg/l)**	**(mg/l)**	**(mg/l)**	**(mg/l)**	**(mg/l)**	**(mg/l)**	**(mg/l)**	**(mg/l)**	**(mg/l)**	**(μmhos/cm)**	**(mg/l)**
P1	8.1	103.5	20.57	26	67.45	0	0	170.8	144	445.41	707	150
P2	8.2	200.1	6.05	20	149.1	0	0	122	196.8	648.9	1030	75
P3	8.4	255.3	29.04	22	188.15	0	18	225.7	249.6	900.9	1430	175
P4	8	331.2	38.72	76	426	0	0	109.8	364.8	1348.2	2140	350
P5	8.1	317.4	29.04	48	337.25	0	0	103.7	360	1165.5	1850	240
P6	7.9	305.9	50.82	100	390.5	0	0	286.7	336	1367.1	2170	460
P7	8	200.1	36.3	80	276.9	0	0	183	240	975.24	1548	350
P8	7.9	920	108.9	300	1544.3	105.3	0	268.4	912	4145.4	6580	1200
P9	8.1	736	200.86	172	1082.8	62.4	0	213.5	1200	3666.6	5820	1260
P10	8.1	1324.8	118.58	224	2165.5	132.6	0	195.2	864	5040	8000	1050
P11	8.1	506	41.14	86	656.75	0	0	183	393.6	1864.8	2960	385
P12	8.1	740.6	108.9	124	1189.3	31.2	0	109.8	624	2973.6	4720	760
P13	8.3	425.5	42.35	70	550.25	0	24	97.6	364.8	1606.5	2550	350
P14	7.9	1094.8	106.48	284	1917	105.3	0	109.8	849.6	4599	7300	1150
P15	7.9	1163.8	94.38	292	1970.3	117	0	122	897.6	4743.9	7530	1120
P16	8.3	680.8	106.48	68	852	11.7	18	195.2	696	2601.9	4130	610
P17	8.8	349.6	38.72	36	418.9	0	24	103.7	283.2	1260	2000	250
P18	8.1	391	88.33	94	667.4	0	0	109.8	408	1808.1	2870	600
P19	8.3	147.2	47.19	48	145.55	0	12	207.4	240	774.9	1230	315
P20	8.5	322	50.82	38	489.9	0	18	122	187.2	1241.1	1970	305
P21	8.8	384.1	9.68	8	347.9	0	30	170.8	211.2	1118.9	1776	60
P22	8.3	368	53.24	46	443.75	0	24	158.6	321.6	1430.1	2270	335
P23	8.4	749.8	96.8	110	1029.5	19.5	18	85.4	748.8	2929.5	4650	675
P24	8.3	1495	116.16	236	2485	179.4	18	48.8	945.6	5632.2	8940	1070
P25	8.3	1035	121	134	1597.5	89.7	18	79.3	825.6	3994.2	6340	835
P26	9.4	1219	54.45	30	1331.3	85.8	102	109.8	897.6	3824.1	6070	300
P27	8.4	16.1	26.62	52	28.4	0	24	152.5	76.8	327.6	520	240
P28	8.4	64.4	19.36	22	35.5	0	12	146.4	86.4	330.75	525	135
P29	8.5	41.4	35.09	20	35.5	0	12	201.3	57.6	342.09	543	195
P30	8.2	285.2	24.2	46	319.5	0	0	164.7	249.6	1030.7	1636	215
**Min**	7.9	16.1	6.1	8.0	28.4	0.0	0.0	48.8	57.6	327.6	520.0	60.0
**Max**	9.4	1495.0	200.9	300.0	2485.0	179.4	102.0	286.7	1200.0	5632.2	8940.0	1260.0
**Ave**	8.3	539.1	64.0	97.1	771.3	31.3	12.4	151.9	474.4	2137.9	3393.5	507.2
**SD**	0.32	415.68	44.81	87.05	701.77	51.52	19.80	56.51	322.67	1599.95	2539.61	374.27

**Table 3:** Calculation of RSC, PI, KR, MH, Na%, SAR and SSP of ground water for 2013 and 2014.

**Well**	**2013**						**2014**							
**ID**	**RSC**	**PI**	**KR**	**MH**	**Na%**	**SAR**	**SSP**	**RSC**	**PI**	**KR**	**MH**	**Na%**	**SAR**	**SSP**
P1	0.2	85.2	1.3	60.0	56.9	3.0	56.9	−0.2	82.31	1.50	56.67	60	3.7	60.0
P2	−5.5	49.8	0.6	57.4	36.5	2.5	36.5	0.5	99.16	5.80	33.33	85.29	10.0	85.3
P3	0.4	89.5	3.2	66.7	76.3	8.6	76.3	0.8	89.20	3.17	68.57	76.03	8.4	76.0
P4	−5.9	71.1	1.9	41.1	65.6	7.3	65.6	−5.2	73.56	2.06	45.71	67.29	7.7	67.3
P5	−3.1	82.2	3.2	38.6	76.0	9.4	76.0	−3.1	81.20	2.88	50.00	74.19	8.9	74.2
P6	−5.3	65.0	1.2	44.2	55.4	5.4	55.4	−4.5	68.75	1.45	45.65	59.11	6.2	59.1
P7	−4.1	64.6	1.1	36.0	53.1	4.4	53.1	−4	66.45	1.24	42.86	55.41	4.7	55.4
P8	−20.5	64.9	1.6	38.7	63.0	11.4	61.7	−19.6	65.78	1.67	37.50	64.02	11.5	62.5
P9	−21.8	59.5	1.3	62.9	57.8	9.1	56.5	−21.7	59.21	1.27	65.87	57.14	9.0	55.9
P10	−18.8	74.1	2.5	41.8	73.1	16.9	71.8	−17.8	75.56	2.74	46.67	74.39	17.8	73.3
P11	−13.5	70.3	2.1	53.3	68.1	11.5	67.7	−4.7	79.91	2.86	44.16	74.07	11.2	74.1
P12	−5.2	77.7	2.6	48.6	72.4	9.8	72.4	−13.4	70.76	2.12	59.21	68.46	11.7	67.9
P13	−21.5	69.7	2.1	37.1	69.1	14.4	67.9	−4.6	77.51	2.64	50.00	72.55	9.9	72.5
P14	−21	71.0	2.3	38.1	70.5	15.2	69.3	−21.2	69.32	2.07	38.26	68.62	14.0	67.4
P15	−9.2	75.2	2.4	66.2	71.2	12.4	70.8	−20.4	71.25	2.26	34.82	70.53	15.1	69.3
P16	−3.2	79.5	2.5	46.7	71.6	8.7	71.6	−8.4	75.09	2.43	72.13	71.02	12.0	70.8
P17	−6.3	83.2	3.8	60.2	79.3	16.6	79.3	−2.5	81.70	3.04	64.00	75.25	9.6	75.2
P18	−1.8	66.6	1.0	54.0	49.6	3.5	49.6	−10.2	63.25	1.42	60.83	58.62	6.9	58.6
P19	−4	77.2	2.3	63.5	70.0	8.3	70.0	−2.5	64.91	1.02	61.90	50.39	3.6	50.4
P20	2.8	105.4	20.6	62.5	95.4	26.1	95.4	−3.5	76.69	2.30	68.85	69.65	8.0	69.7
P21	−16.5	75.3	2.8	52.2	74.8	16.7	73.5	2.6	102.64	13.92	66.67	93.30	21.6	93.3
P22	**−**12.4	71.8	2.2	60.1	69.2	11.8	68.8	−3.3	77.59	2.39	65.67	70.48	8.7	70.5
P23	**−**21.9	75.9	3.0	40.9	76.0	20.1	74.7	−11.5	73.28	2.41	59.26	71.03	12.5	70.7
P24	2.2	95.5	11.1	74.0	92.2	35.2	91.8	−20	76.27	3.04	44.86	76.48	19.9	75.2
P25	**−**11.3	70.5	2.0	51.3	66.5	10.7	66.2	−14.8	74.78	2.69	59.88	73.91	15.6	72.9
P26	**−**1.5	49.5	0.2	41.9	17.3	0.6	17.3	−0.8	92.10	8.83	75.00	90.20	30.6	89.8
P27	0.7	84.5	1.4	50.0	57.9	3.0	57.9	−1.5	41.48	0.15	45.83	12.73	0.5	12.7
P28	0.1	67.6	0.5	50.0	34.5	1.4	34.5	0.1	79.08	1.04	59.26	50.91	2.4	50.9
P29	**−**1.5	85.3	3.1	35.0	75.6	8.8	75.6	−0.2	63.45	0.46	74.36	31.58	1.3	31.6
P30	**−**0.8	57.4	0.2	47.1	19.0	0.6	19.0	−1.6	84.09	2.88	46.51	74.25	8.5	74.3
**Min**	**−**21.9	49.5	0.21	35	1.2	17.31	0.61	17.31	41.48	0.15	33.33	12.73	0.45	12.73
**Max**	2.8	105.39	20.63	74	83.9	95.38	35.23	95.38	102.64	13.92	75	93.30	30.60	93.30
**Ave**	**−**7.67	73.84	2.88	50.67	27.60	63.80	10.44	63.44	75.21	2.79	54.81	66.56	10.38	66.23
**SD**	8.14	12.24	3.86	10.73	24.06	18.07	7.66	17.95	11.94	2.64	12.23	15.88	6.37	15.82

**Table 4:** Quality of ground water samples from rural area in Neyshabour County for drinking purpose (BIS standard).

**Parameter**	**Desirable**	**2013 Year**		**2014 Year**	
	**limit**	**Samples (%)**		**Samples (%)**	
		**Within limits**	**Exceed limits**	**Within limits**	**Exceed limits**
**pH**	6.5–8.5	93.3	6.7	85.3	14.7
**EC**	300 (μmhos/cm)	0	100	0	100
**TDS**	500 (mg/L)	16.7	83.3	13.3	86.7
**T H**	200 (mg/L)	23.3	76.7	20	80
**SO4**^**−2**^	200 (mg/L)	20	80	20	80
**Cl**^**−**^	250 (mg/L)	26.7	73.3	23.3	76.7
**Ca**^**+2**^	75 (mg/L)	50	50	53.3	46.7
**Mg**^**+2**^	30 (mg/L)	30	70	26.7	73.3
**Na**^**+**^	200 (mg/L)	26.7	73.3	16.7	83.3

**Table 5:** Classification of Ground water sample for irrigation use on the basic of EC, SAR, RSC, KR, SSP, PI, MH, Na%, T.H.

**Parameters**	**Range**	**Water class**	**Samples (%)**	
			**2013 Year**	**2014 Year**
**EC**	<250	Excellent	Nil	Nil
	250–750	Good	16.7	13.3
	750–2250	Permissible	36.7	36.7
	>2250	Doubtful	46.7	50
**SAR**	0–10	Excellent	56.7	60
	10–18	Good	33.3	30
	18–26	Doubtful	3.3	6.7
	>26	Unsuitble	6.7	3.3
**RSC**	<1.25	Good	93.3	96.7
	1.25–2.5	Doubtful	93.3	Nil
	>2.5	Unsuitble	93.3	3.3
**KR**	<1	Suitble	16.7	6.7
	1–2	Marginal suitble	26.7	26.7
	>2	Unsuitble	56.7	66.7
**SSP**	<50	Good	16.7	6.7
	>50	Unsuitble	83.3	93.3
**PI**	>75	Class-I	46.7	53.3
	25–75	Class-II	53.3	46.7
	<25	Class-III	Nil	Nil
**MH**	<50	Suitble	53.3	46.7
	>50	Harmful and Unsuitble	46.7	53.3
**Na%**	<20	Excellent	6.7	3.3
	20–40	Good	6.7	3.3
	40–60	Permissible	20	23.3
	60–80	Doubtful	60	60
	>80	Unsuitble	6.7	10
**T.H**	<75	Soft	3.3	6.7
	75–150	Moderately Hard	6.7	6.7
	150–300	Hard	26.7	23.3
	>300	Very Hard	63.3	63.3

## 2 Experimental design, materials and methods

### 2.1 Study area description

Nishabur County is located in Khorasan-e Razavi province in northeastern Iran and the county capital is the city of Nishabur. Nishabur County is located in a fertile plain at the foot of the Binalud Mountains. In this study, the three important wheat production regions of Bar, Barzanon and Eshghabad were selected as sampling points (Fig. 1).

### 2.2 Determination of the physicochemical parameters concentration

To assess the physicochemical parameters, 30 water samples were collected during 2013 and 2014 from villages in Nishabur County (Fig. 1). Twelve parameters that are characteristic of drinking water were measured. The water samples from all observation wells were stored in a plastic 1-liter container for detailed chemical analysis. These containers were washed thoroughly with distilled water and dried before being filled with the water samples. To obtain a composite sample, they were collected after the well was subjected to pumping for 5–10 min. The experiments were done using system and titrimetric testing for temporary and permanent hardness, calcium, magnesium and chloride levels. All sampling steps and data analysis was performed according to standard methods for water and wastewater [1], [2], [3], [4], [5] (Table 6).

**Table 6:** Summary of water quality indices in present study.

**Indices**	**Formula**
Residual Sodium Carbonate (RSC)	RSC=(CO_3_^2−^+HCO_3_^−^)+(Ca^2+^+Mg^2+^)
Permeability Index (PI)	$\mathrm{PI}=\frac{\mathrm{Na}+K+\sqrt{\mathrm{HCO}3}}{\mathrm{Ca}+\mathrm{Mg}+\mathrm{Na}+K}\times 100$
Kelly's Ratio (KR)	$\mathrm{KR}=\frac{\mathrm{Na}}{\mathrm{Ca}+\mathrm{Mg}}$
Magnesium Hazard(MH)	$\mathrm{MH}=\frac{\mathrm{Mg}}{\mathrm{Ca}+\mathrm{Mg}}\times 100$
Sodium percentage (Na %)	$\mathrm{Na},\%=\frac{\mathrm{Na}+K}{\mathrm{Ca}+\mathrm{Mg}+\mathrm{Na}+K}\times 100$
Sodium Adsorption Ratio (SAR)	$\mathrm{SAR}=\frac{\mathrm{Na}}{\sqrt{(\mathrm{Ca}+\mathrm{Mg})/2}}\times 100$
Soluble Sodium Percentage (SSP)	$\mathrm{SSP}=\frac{\mathrm{Na}}{\mathrm{Ca}+\mathrm{Mg}+\mathrm{Na}}\times 100$
