# Supplementary material for: Data on water quality index for the groundwater in rural area Neyshabur County, Razavi province, Iran
Source: Data Brief. 2017 Nov 1;15:901–7. doi: 10.1016/j.dib.2017.10.052 (PMC5681322; doi:10.1016/j.dib.2017.10.052)
Supplement: Supplementary file 1 — Transparency document [file mmc1.docx]

Competing interests

We affirm that this article is the original work of the authors and have no conflict of interest to declare.

Authors’ contributions:

All authors reviewed the manuscript. All authors participated in writing the manuscript.

This study funded by Neyshabur University of Medical Sciences the authors are grateful for the financial support provided by the mentioned center.
